# Domain-specific interactions between MLN8237 and human serum albumin estimated by STD and WaterLOGSY NMR, ITC, spectroscopic, and docking techniques

**DOI:** 10.1038/srep45514

**Published:** 2017-03-30

**Authors:** Hongqin Yang, Jiuyang Liu, Yanmei Huang, Rui Gao, Bin Tang, Shanshan Li, Jiawei He, Hui Li

**Affiliations:** 1 College of Chemical Engineering, Sichuan University, Chengdu 610065, People’s Republic of China; 2 School of Life Sciences, University of Science and Technology of China, Hefei 230026, People’s Republic of China

## Abstract

Alisertib (MLN8237) is an orally administered inhibitor of Aurora A kinase. This small-molecule inhibitor is under clinical or pre-clinical phase for the treatment of advanced malignancies. The present study provides a detailed characterization of the interaction of MLN8237 with a drug transport protein called human serum albumin (HSA). STD and WaterLOGSY nuclear magnetic resonance (NMR)-binding studies were conducted first to confirm the binding of MLN8237 to HSA. In the ligand orientation assay, the binding sites of MLN8237 were validated through two site-specific spy molecules (warfarin sodium and ibuprofen, which are two known site-selective probes) by using STD and WaterLOGSY NMR competition techniques. These competition experiments demonstrate that both spy molecules do not compete with MLN8237 for the specific binding site. The AutoDock-based blind docking study recognizes the hydrophobic subdomain IB of the protein as the probable binding site for MLN8237. Thermodynamic investigations by isothermal titration calorimetry (ITC) reveal that the non-covalent interaction between MLN8237 and HSA (binding constant was approximately 10^5^ M^−1^) is driven mainly by favorable entropy and unfavorable enthalpy. In addition, synchronous fluorescence, circular dichroism (CD), and 3D fluorescence spectroscopy suggest that MLN8237 may induce conformational changes in HSA.

Aurora A kinase (AAK) belongs to a family of oncogenic serine/threonine protein kinases that are associated with centrosome maturation and separation, thereby regulating spindle assembly and stability^1^. Numerous studies have found that AAK is strongly expressed in a variety of human hematological malignancies and solid tumors, including bladder, breast, colorectal, upper gastrointestinal, head and neck, lung, ovarian, pancreatic, and prostate cancer^2^,^3^,^4^,^5^. AAK is a new inhibition target for antitumor drugs because of its major function in cell mitosis. In recent years, several selective AAK inhibitors have been invented domestically and abroad and showed good therapeutic effect^6^.

Alisertib (4-[[9-Chloro-7-(2-fluoro-6-methoxyphenyl)-5H-pyrimido[5,4-d][2] benzazepin-2-yl]amino]-2-methoxybenzoic acid), also called MLN8237, is an investigational, orally administered, and selective AAK inhibitor that has exhibited broad-spectrum anticancer activity in pre-clinical and clinical studies^1^,^7^. Several studies demonstrated that MLN8237 disrupts cell cycle kinetics, impairs growth, induces a cellular phenotype consistent with AAK inhibition, and triggers apoptosis in chronic myelogenous leukemia cell lines^5^,^8^. Kelly *et al*.^8^ confirmed that MLN8237 can significantly inhibit AAK auto-phosphorylation and histone–H3 phosphorylation and increase the anticancer activity of the standard agent nilotinib through combined medication scheme. Qi *et al*.^9^ demonstrated that MLN8237 with docetaxel may represent a novel therapeutic strategy that could inhibit the growth of tumor cells and enhance survival better than signal agent therapy could. MLN8237 molecules exhibit low solubility; thus, the drug was originally developed as an orally administered drug for initial phase I and II clinical studies^5^. After oral administration, MLN8237 is mainly circulated in plasma and reaches the target tissues by binding to serum albumin. The therapeutic efficacy of drugs is directly related to free drug concentration in blood plasma; thus, the binding ability towards plasma proteins is one of the critical pharmacokinetic parameters in drug delivery^10^.

Human serum albumin (HSA) is the most widely distributed protein in human blood plasma. It binds to a variety of endogenous and exogenous compounds with moderate to high association constant (10^4^–10^6^ M^−1^)^11^ and also acts as a transporter for drugs and other organic molecules to their targets^12^. Previous structural biological studies reveal that HSA is a globular protein that consists of a single polypeptide chain of 585 amino acid residues and is composed of three homologous helical domains (named I, II, and III)^13^. Each domain is known to be made up of two separate helical subdomains (named A and B) connected by a random coil^14^. Binding mainly occurs in subdomains IIA and IIIA, known as Sudlow’s sites I and II. Most previous reports recommend that heterocyclic compounds with a diffused negative charge at the center of the molecule often acts as a subdomain IIA specific drug^15^,^16^,^17^,^18^. In addition, the subdomain IIIA generally binds with stick-like aromatic carboxylic acids, which are usually referred to as indole–benzodiazepine in the binding region^16^. In addition to these possibilities, drugs that bind to other subdomains, including subdomains IA, IB, IIB, and IIIB, exist. That binding may strongly affect the absorption of numerous drugs, apparent drug distribution volume, and elimination rate. Therefore, understanding the mechanism of interaction of drugs with serum albumin will aid in the interpretation of drug metabolism and transport mechanism.

Yang *et al*.^19^ previously reported that MLN8237 is highly protein bound (97%), and pharmacokinetic (PK) parameters in preclinical models suggested favorable PK in humans. The majority of the recently published studies on this drug are primarily aimed at preclinical metabolism, pharmacokinetics, and efficacious dose^5^,^19^,^20^. Alam *et al*.^21^ recently reported the interaction of the newly approved tyrosine kinase inhibitor nintedanib with HSA, confirming the existence of a weak interaction between the inhibitor and HSA. However, to the best of our knowledge, the interaction between threonine kinase inhibitor MLN8237 and HSA has not been studied. In the present study, the binding of MLN8237 with HSA was investigated using various analytical techniques and molecular docking, which thoroughly extend our knowledge of the complex mechanisms involved in the drug–HSA binding process. Saturation transfer difference (STD) and water–ligand observed through gradient spectroscopy (WaterLOGSY) nuclear magnetic resonance (NMR) experiments were first conducted to validate the fine details of the MLN8237-HSA interaction. Second, STD and WaterLOGSY NMR competition binding experiments, with warfarin sodium and ibuprofen as spy molecules, were performed to assess the drug molecule possible binding sites on the HSA binding pocket. Subsequently, the experimental results were validated by molecular docking, as well as proposed a three-dimensional (3D) model of the complex mechanisms. Finally, the thermodynamics of the binding of MLN8237 to HSA were investigated by using isothermal titration calorimetry (ITC), and the consequent protein conformation change was monitored using synchronous fluorescence, circular dichroism (CD) and 3D fluorescence spectroscopy. In the present study, the investigation of the binding nature of MLN8237 with HSA is of interest, is helpful in understanding the mechanism of antitumor and anticancer activities of the drug, and can provide support for the continued clinical investigation of the drug.

## Results and Discussion

### STD and WaterLOGSY NMR studies

The STD and WaterLOGSY NMR methods are extremely useful techniques to probe and understand weak interactions between small molecules and macromolecules at the molecular level^22^,^23^,^24^,^25^. In the case of STD, the saturation of protein is spread onto the ligand owing to the binding and then the signals of the bound-ligand is identified. In WaterLOGSY, the bulk water is selectively saturated. Magnetisation is transferred from water to both free ligands and protein-bound ligands via the protein. The signals allows distinguishing binder and non-binder ligands^24^. In the following section, STD and WaterLOGSY NMR spectroscopic binding investigations were applied to observe the AAK inhibitor MLN8237–HSA complex system in detail.

**Figure 1:**
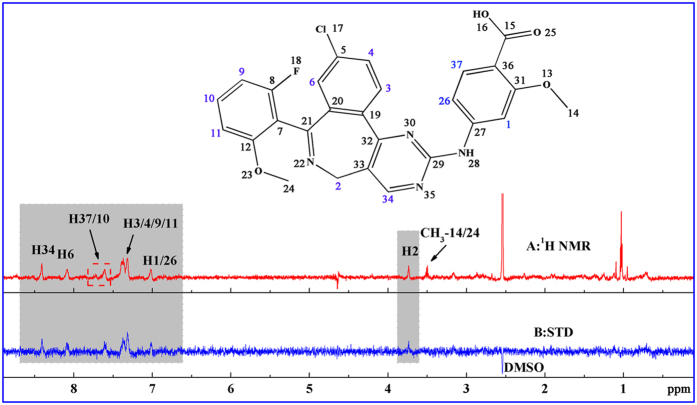
^1^H NMR spectrum (**A**) and STD NMR spectrum (**B**) of MLN8237 in the presence of HSA in 40:1 ratio as recorded with a Watergate scheme for solvent suppression (50% H_2_O/50% D_2_O PBS, pH 7.4 at 298 K). [MLN8237] = 400 μM, [HSA] = 10 μM.

In the first experiment, the ^1^H NMR spectrum of MLN8237 with protein and the corresponding STD NMR spectrum are presented in Fig. 1, and the compound chemical shift values are fully assigned in Fig. 1A. Clear STD signals attributed to MLN8237 in a non-ambiguous binding manner to HSA appeared on the spectrum. In the STD NMR spectrum, the signal intensity of each proton peak reflects not only the relative amount of saturation received by the particular proton from the macromolecule through magnetization transfer but also the relative proximity to the macromolecule. Thus, a relative STD value for each proton of MLN8237 should be obtained on the basis of every peak area integral value. However, the relative STD effect was not calculated because of numerous overlapping peaks of STD signals of the compound present in the phosphate buffer solution (PBS), particularly, H3, H4, H9, and H11 aromatic protons. The overlapping peaks increased the integral errors. However, MLN8237 was indeed bound to HSA.

A WaterLOGSY NMR experiment was also conducted with the same drug/protein concentration ratio in the STD experiment to further support the STD results. In this experiment, the presence of positively phased signals of the ligand molecules relative to DMSO signal in the presence of macromolecules is attributed to interaction. The ^1^H NMR spectrum of the compound is displayed in Supplementary Fig. S1a for reference. As shown in Supplementary Fig. S1b, direct interaction between MLN8237 and HSA is evident from the positively phased signals of the compound in the presence of 10 μM HSA. The WaterLOGSY result showed excellent agreement with the previous STD experiments.

### Identification of binding site study on HSA

Once the binding of MLN8237 to HSA is determined, the location of the possible protein-binding sites where the interaction occurs should also be addressed. To accomplish this goal, a competitive spy molecule with a defined binding site in the protein receptor is necessary to form a comparison with the unknown molecule so that the corresponding binding site can be located. In this study, STD and WaterLOGSY NMR competition displacement experiments were conducted under simulated physiological conditions. In general, titration of a competitive spy molecule in a solution containing an unknown ligand can either reduce or wipe out the signal intensities of that ligand in the STD spectrum when the spy is a better binder to the specific site. While the signals in the WaterLOGSY are reducing or becoming negative. On the contrary, if this ligand itself possesses a better binder than the spy, adding the spy does not affect the signal intensity of the respective ligand proton signals. Then, outside these possibilities, none of the signals will be affected if the spy molecule and unknown ligand bind with different sites^23^,^26^.

**Figure 2:**
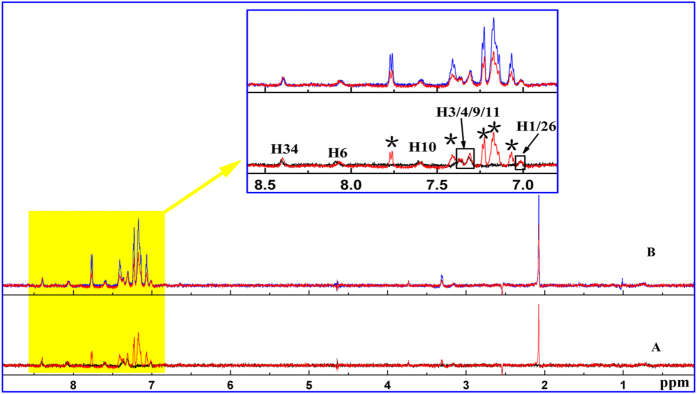
STD competition binding experiments recorded at a 700 MHz spectrometer in 50% H_2_O/50% D_2_O PBS, pH 7.4 at 298 K. (**A**) Overlaid view of STD spectra of the system MLN8237 (400 μM)/HSA (10 μM) (black solid line) and MLN8237 (400 μM)/warfarin sodium (480 μM)/HSA (10 μM) (red solid line); (**B**) Overlaid view of STD spectra of the system MLN8237 (400 μM)/warfarin sodium (480 μM)/HSA (10 μM) (red solid line) and MLN8237 (400 μM)/warfarin sodium (1200 μM)/HSA (10 μM) (blue solid line). The proton signals of warfarin sodium are indicated by *.

To complete the experiments, warfarin sodium and ibuprofen were used as spy molecules to estimate the specific binding site of MLN8237. Warfarin sodium, an effective chemoprophylaxis agent against thromboembolic disease, is used as a reference spy that preferably binds to site I of HSA^27^. Ibuprofen, a nonsteroidal anti-inflammatory drug, is recognized as a typical binding spy for site II of HSA^24^. First, the STD binding experiments at a constant MLN8237/HSA molar ratio of 40:1 (400 μM MLN8237 and 10 μM HSA) were performed as control. Subsequently, different STD-competition binding experiments were measured by gradually adding warfarin sodium at two different concentrations (480 and 1200 μM) while keeping MLN8237 and HSA constant; the results are shown in Fig. 2. At the two concentrations, the STD signals of MLN8237 and warfarin sodium protons were observed simultaneously. As shown in the magnification analysis of the aromatic region, the STD signal intensities of MLN8237 protons did not reduce the increasing amount of warfarin sodium, whereas the STD signal intensities for warfarin sodium protons increased. Considering the competition results, we can conclude that MLN8237 does not target the same binding site (site I) as warfarin sodium.

Similarly, further STD competition experiments for the hunt of site II were performed with ibuprofen as a spy molecule. Different STD competition experiments with varying concentrations (480 and 1200 μM) of ibuprofen showed a similar behavior in the STD spectra with the results of warfarin sodium. As indicated in Supplementary Fig. S2, in the STD signals of MLN8237, each proton shows that the STD signal intensities of MLN8237 protons remained constant despite the increasing molar excess of ibuprofen. The results further suggest that MLN8237 does not target the selected site II of HSA.

Similar results are obtained with WaterLOGSY competition experiments versus warfarin sodium and ibuprofen; the data are provided as a supplementary file (see Supplementary Figs S3 and S4). The WaterLOGSY response to the addition of spy molecules shows no decrease in the MLN8237 WaterLOGSY signal intensities, thereby confirming the STD findings that the binding of MLN8237 does not locate preferentially within sites I and II of HSA. We can presume from the aforementioned competition studies that none of the two spy molecules (warfarin sodium and ibuprofen) compete with MLN8237 for the same binding site despite showing binding to HSA, thereby indicating interaction toward other binding loci in the hydrophobic cavities of the protein.

Typically, in a WaterLOGSY experiment, a 90% H_2_O/10% D_2_O mixture (or even 95% H_2_O/5% D_2_O) is used because D_2_O is a poor relaxation agent and is very poor in ‘transferring magnetisation’ to other molecules via NOE. In order to further validate the rationality of our experimental setup, the STD and WaterLOGSY experiments were performed in 90% H_2_O/10% D_2_O PBS, pH 7.4 at 298 K. The results are displayed in Supplementary Figs S5 and S6, and S7. As shown in Supplementary Fig. S5, although the STD signals of both types of mixtures were same, the quality of STD spectra and signal-noise ratio are better in the 50% H_2_O/50% D_2_O mixture. For the STD and WaterLOGSY NMR competition displacement experiments, the results obtained in the 90% H_2_O/10% D_2_O mixture show no decrease in the MLN8237 STD and WaterLOGSY signal intensities (see the Supplementary Figs S6 and S7). The results agree well with those found in our above experiments, thereby justifying the choice of such an unusual experimental setup (a 50% H_2_O/50% D_2_O mixture).

### Molecule docking analysis

**Figure 3:**
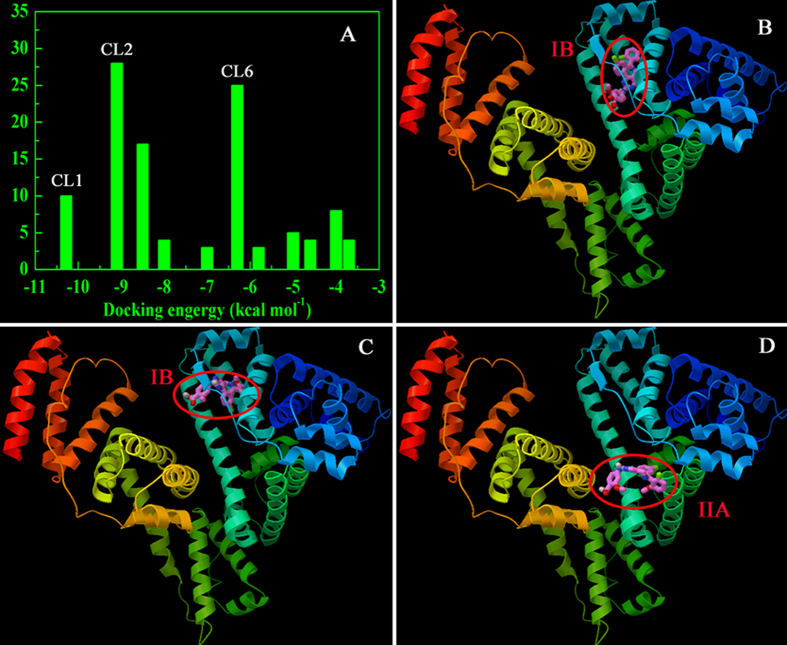
Cluster analyses of the AutoDock docking runs of MLN8237-HSA system (**A**) and binding interaction regions corresponding to cluster 1 (**B**), cluster 2 (**C**) and cluster 6 (**D**).

Further observations were conducted through a molecular docking study based on a blind docking simulation to ascertain the possible binding locus between MLN8237 and HSA. Cluster analysis was performed using a rmsd tolerance of 2.0 Å. A total of 11 multimember conformational clusters were gathered from 100 docking runs (Fig. 3A). From Fig. 3B, the first cluster (CL1, 11 out of 100 conformations) for the hydrophobic subdomain IB of HSA was found to be the lowest on the energy scale. Hence, CL1 was the most energetically favorable conformational cluster, possessing an estimated docking energy of about −10.28 kcal M^−1^. For the most populated cluster (CL2), about 28 distinct conformational models were observed. The result still revealed that the drug bound preferentially to the subdomain IB of HSA (Fig. 3C). Whereas for Fig. 3D, the analysis of the docking disposition of the conformers belonging to CL6 supported the possible interaction of MLN8237 into the subdomain IIA in site I of HSA. Although about 25 distinct conformational clusters were obtained for CL6, it was not the highest populated cluster and the most energetically favorable. According to the docking principle of rationality, the lowest binding energy conformation of ligand-protein complexes was used for further docking analysis. Therefore, the subdomain IB of the protein was considered as the favorable binding region for the drug. The best docked result was then used for further binding orientation analysis and is displayed in Supplementary Fig. S8. The observation shows that MLN8237 was surrounded by hydrophobic residues PHE134, LEU135, TYR138, LEU154, TYR161, SER193, as well as charged/polar residues ILE142, HIS 146, PHE157, ALA159, PHE165, GLY189, LYS190. The results evince the binding phenomenon to be mainly governed by hydrophobic forces with a significant contribution from electrostatic interactions.

### ITC measurements

**Figure 4:**
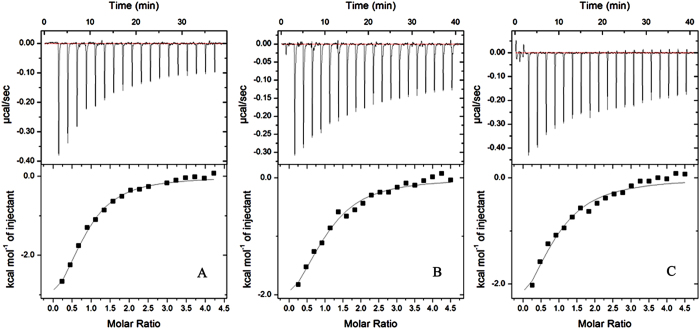
HSA calorimetric titration curves along three different temperature levels at pH 7.4. (**A**–**C**) represent the ITC profiles of the MLN8237–HSA system at 298, 304, and 310 K, respectively.

After the interaction and specific binding site between MLN8237 and HSA was identified, the subsequent step was to estimate the magnitudes of binding affinities and associated thermodynamic parameters. The ITC technique can simultaneously measure the thermodynamic equilibrium constant (*K*), which is closely related to free energy variation (Δ*G*), enthalpy (Δ*H*), entropy (Δ*S*) variations, and binding stoichiometry (*N*)^28^,^29^. Three representative calorimetric titration profiles of MLN8237 to HSA at 298, 304, and 310 K at pH 7.4 are exhibited in Fig. 4A,B and C, respectively. Each peak in the binding isotherm corresponds to a single injection of MLN8237 into the HSA solution. As shown in Fig. 4, the profile reveals an exothermic characteristic. Each peak fitted according to the independent binding model results in the corresponding thermodynamic parameters (including *K*, Δ*H*, and *N*). The changes in Δ*G* and Δ*S* at three different temperature levels were calculated using the following equations^28^,^30^:


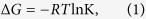



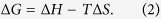


As shown by the data in Table 1, only one binding event (*N* ≈ 1) occurred at the three temperature levels, indicating that one type of complexation is formed exclusively. Negative values of Δ*H* and positive values of Δ*S* at all studied temperature levels suggest that the binding of MLN8237 with HSA is predominantly driven by entropy. However, the same results were reported for several other protein–ligand complexes by using ITC or fluorescence spectroscopic methods^31^,^32^. The process is ubiquitous throughout protein–ligand interactions. Ross and Subramanian^33^ proved that negative Δ*H* and positive Δ*S* values indicate the involvement of electrostatic attraction and hydrophobic interaction in the formation of the protein–ligand complex. The binding constant, Δ*H*, and Δ*S* terms increased with the increase in temperature, thereby keeping the Δ*G* virtually constant. This result shows that temperature affects the binding between MLN8237 and HSA. The negative sign for Δ*G* suggests the spontaneity of the binding of MLN8237 with HSA.

The results obtained by ITC analysis were compared with the binding constant from fluorescence measurements based on the method we reported before^34^. From Supplementary Fig. S9, it can be seen that there are no fluorescence emission for MLN8237 at the range measured, and a significant decrease in the fluorescence intensity of HSA was observed, when various MLN8237 concentrations were added into a fixed concentration of HSA. The results indicated that the binding of MLN8237 to HSA quenched the intrinsic fluorescence of the tryptophan residues. The binding constant of bound MLN8237 to HSA was determined by plotting the double logarithm regression curve of the fluorescence data using the “modified” Stern-Volmer equation and calculated to be (10.15 ± 0.21) × 10^4^ M^−1^, which is consistent with the ITC result.

### Conformation investigations

#### Synchronous fluorescence measurements

Synchronous fluorescence spectroscopy, which was introduced by Lloyd in 1971^35^,^36^, is a widely accepted technique developed to probe the effect of ligands on the microenvironment of amino acid residues of protein. The possible shift in the position of the maximum emission wavelength of the amino acid residues corresponds to the changes in polarity in the vicinity of the chromophore molecule, revealing the conformational change in HSA^37^,^38^. Miller^39^ proposed the long-standing theory that when Δλ was stabilized at 15 and 60 nm, the spectral characteristic information of tyrosine (Tyr) and tryptophan (Trp) residues of the protein were respectively observed. A blue shift of *λ*_max_ means that the amino acid residues are located in a hydrophobic environment, whereas a red shift of *λ*_max_ implies that the amino acid residues are in a polar environment^38^,^40^.

**Figure 5:**
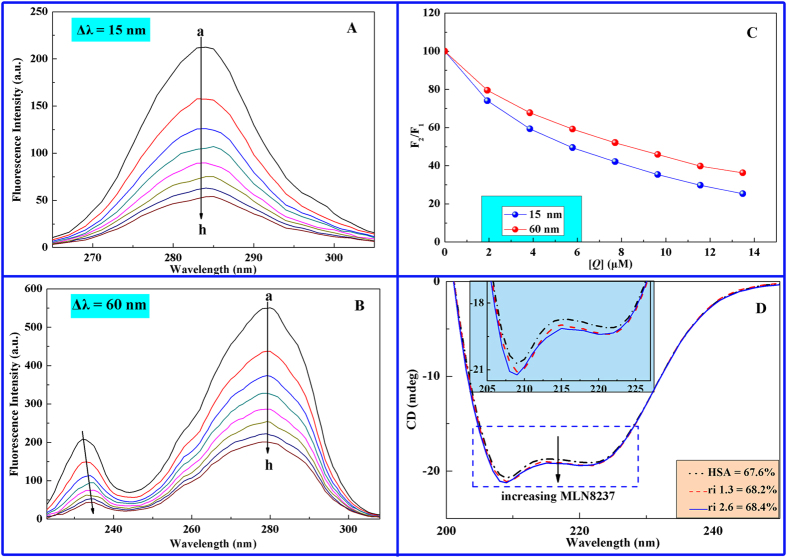
Synchronous fluorescence spectra of HSA-MLN8237 system. (**A**) Δ*λ* = 15 nm; (**B**) Δ*λ* = 60 nm; (**C**) quenching degree of Tyr and Trp, where *F*_1_ and *F*_2_ are the fluorescence intensities of HSA without and with MLN8237, respectively; *C*_HSA_ = 2 μM, C_MLN8237_, a → h: 0, 1.927, 3.854, 5.781, 7.708, 9.635, 11.562, 13.489 (*T* = 298 K); and (**D**) CD spectra of HSA in the absence and presence of MLN8237; C_HSA_ = 1.8 μM and C_MLN8237 _=_ _0, 2.31, 4.62 μM at 298 K.

The synchronous fluorescence spectra of HSA upon addition of the drug molecule at Δ*λ* = 15 and 60 nm are presented in Fig. 5. The decrease in fluorescence intensities of both Tyr and Trp on the successive addition of MLN8237 indicates the occurrence of emission quenching during interaction between MLN8237 and HSA. As shown in Fig. 5A, when Δ*λ* was set to 15 nm, the maximum excitation wavelength presented a red shift of 3 nm (282 nm → 285 nm) in the system, whereas no shift was observed in Fig. 5B when Δ*λ* was 60 nm. The red-shift effects suggested that binding between MLN8237 and HSA increases the polarity around the Tyr residues and decreases hydrophobicity, whereas the Trp residues were minimally affected. In Fig. 5C, the curve of Δ*λ* = 15 nm is lower than that of Δ*λ* = 60 nm, demonstrating that Tyr performs an important role during fluorescence quenching of HSA by MLN8237. Therefore, we can conclude from synchronous fluorescence experiments that MLN8237 induces the change in the microenvironment of amino acid residues in HSA.

#### CD studies

CD is considered as an important tool to elucidate the structure of polypeptides and characterize the secondary structures of proteins^24^,^34^. As shown in Fig. 5D, the CD spectrum of free HSA exhibits two negative Cotton effects in the UV region, that is, at 208 and 222 nm, which are characteristic features of the α-helix structure^31^. The intensities of the negative bands decreased upon addition of MLN8237 without a change in peak positions and shapes, suggesting that the structure of HSA is predominantly an α-helix as well. The α-helical content of free and combined HSA were calculated at 222 nm using the following equations^31^:


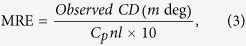



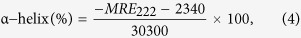


where MRE is the mean residue ellipticity in deg cm^2^ dmol^−1^, *C*_*p*_ is the molar concentration of protein, *n* is the number of amino acid residues (585 for HSA), and *l* is the length of the light path (0.1 cm). The results exhibit a slight increase in the α-helical structure from 67.6% to 68.4% at a molar ratio of MLN8237 to HSA 2.6:1. The increase in α-helical content evidently indicates the binding of MLN8237 to HSA, thereby causing an increase in band intensity in the CD spectra. Ma *et al*.^41^ believed that the increase could be due to the binding of the carboxyl group of the drug with the amino acid residues of the main polypeptide chain of HSA to stabilize the helical structure. Thus, the aforementioned results indicate that the occurrence of an interaction between MLN8237 and HSA causes conformational changes at the secondary structure level of HSA.

#### 3D fluorescence measurements

**Figure 6:**
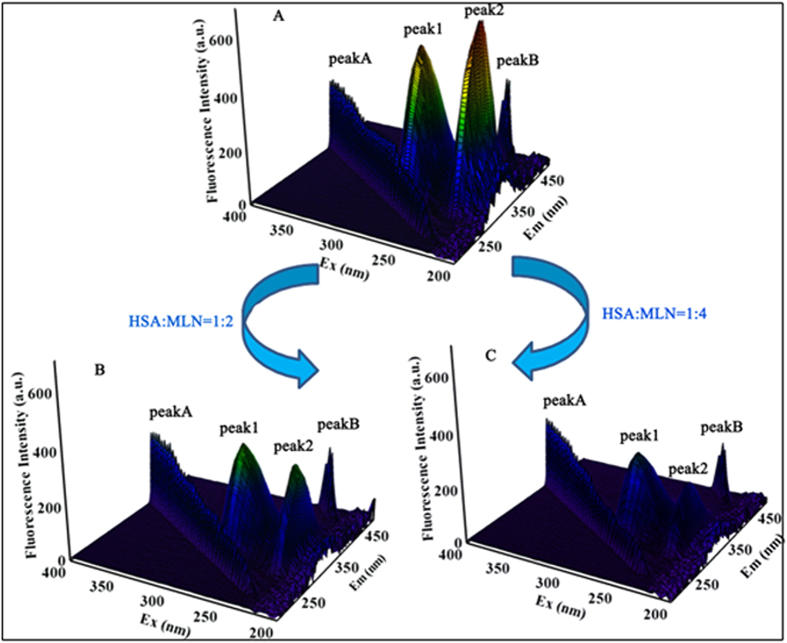
(**A**) 3D fluorescence spectra of HSA alone, (**B**) HSA:MLN8237 1:2 complex, and (**C**) HSA:MLN8237 1:4 complex. The concentration of protein was 2 μM, whereas the concentrations of MLN8237 were 4 and 8 μM.

3D fluorescence spectra were recorded to provide a complete interpretation of the conformational and structural changes in proteins that interact with drugs. As shown in Fig. 6, the 3D fluorescence spectra of HSA were investigated by comparing spectral changes in the absence and presence of MLN8237. The corresponding spectral characteristic parameters elucidated are presented in Table 2. Four typical peaks marked as A, B, 1, and 2 could be easily observed in Fig. 6. Peaks A and B are the first-order Rayleigh scattering peak (*λ*_em_ = *λ*_ex_) and second-order scattering peak (*λ*_em_ = 2*λ*_ex_), respectively^34^. Peak 1 (*λ*_ex_ = 280 nm and *λ*_em_ = 335 nm) is related to the spectral characteristic of Tyr and Trp residues involving π → π* transition and reflects the polarity of the HSA microenvironment^42^,^43^. Peak 2 (*λ*_ex_ = 225 nm and *λ*_em_ = 328 nm) mainly exhibits the spectral behavior of the polypeptide chain backbone structure, and its fluorescence intensity is correlated with the secondary structure of the protein^34^,^38^. Table 2 and Fig. 6 show that the fluorescence intensity of peaks A, 1, and 2 are decreased significantly by increasing the additional MLN8237, along with an increase in the magnitude of Stokes shift of 55 → 56 nm for peak 1 and 103 → 107 nm for peak 2. The maximum emission wavelengths of peak 1 showed a slight red shift following the addition of MLN8237, indicating the alteration of the polar environment of the Tyr or Trp residues of HSA. The results were in good agreement with the synchronous fluorescence spectroscopy results. Meanwhile, the differences in peak 2 confirmed that the binding between HSA and MLN8237 induced the change in the polypeptide backbone structures of the protein change. In combination with the synchronous fluorescence results, this finding reveals that the drug induces changes in the polarity of the microenvironment and the conformation of HSA.

## Conclusions

In this study, direct NMR spectroscopy methods, such as STD and WaterLOGSY assay, molecular docking, ITC, and conventional spectroscopy methods have been applied to investigate the interaction between MLN8237 and HSA. STD and WaterLOGSY results revealed that MLN8237 can bind to the hydrophobic cavities of HSA in the buffer solution. Furthermore, the strategic combination of STD competition binding experiments and WaterLOGSY competition binding experiments has enabled us to deduce that MLN8237 does not compete for the same binding site with two defined HSA spy molecules, such as warfarin sodium and ibuprofen. A stereo model of the formed complex between MLN8237 and HSA has been proposed by docking simulation, and the docking results are in good agreement with the NMR competition binding experiments. Data from ITC experiments suggest the spontaneity of the binding of MLN8237 with HSA, and the stoichiometric binding number *N* approximately equals to 1. Based on the molecular docking and ITC, hydrophobic forces play a major role in the binding of MLN8237 to HSA, whereas other forces, such as electrostatic interactions are also present. The MLN8237-induced microenvironmental and conformational changes of HSA were ultimately established using synchronous fluorescence, CD, and 3D fluorescence spectral studies. In summary, the combination of these techniques leads to a complete evaluation of the binding profiles between the selected AAK inhibitor MLN8237 and HSA.

## Experimental Section

### Materials

HSA (approximately 99% and fatty acid-free) was purchased from Sigma Chemical Company (USA), and MLN8237 was purchased from Selleck (USA). Warfarin sodium, ibuprofen, deuterium oxide (D_2_O, 99.9% purity), and dimethyl sulfoxide-d6 (DMSO-d6) were purchased from J&K Scientific Ltd. (China). The HSA stock solution was prepared in 0.1 M PBS at pH 7.4. The MLN8237 stock solution was also prepared by dissolving an appropriate amount of the drugs in DMSO. All reagents were of analytical reagent grade, and thrice-distilled water was used throughout the experiment.

### Procedure

#### Nuclear magnetic resonance (NMR) spectroscopy

All NMR spectra were obtained and recorded on an Agilent (Varian) INOVA-700 spectrometer operating at 298 K. This spectrometer was equipped with a 96-well autosampler and CryoProbe with inverse detection and linear pulsed gradient on the *z*-axis. STD and WaterLOGSY experiments were conducted at a molar ratio of 40:1 (drug/protein) with 50% H_2_O/50% D_2_O PBS buffer and 90% H_2_O/10% D_2_O PBS buffer. The final concentration of HSA in the NMR spectroscopy tube was 10 μM. Competition studies were performed with warfarin sodium and ibuprofen, namely, two known binders of site I and site II of HSA, respectively^24^,^27^. Different concentrations of warfarin sodium or ibuprofen ([warfarin sodium/ibuprofen]/[MLN8237] = 0.0, 1.2, 3.0) were added into the MLN8237-HSA solution, which was used to analyze the effect of warfarin sodium or ibuprofen on the MLN8237-HSA system, so as to determine the binding site of MLN8237.

In the STD experiments, a train of soft Gaussian-shaped pulses with 50 ms length was applied and separated by 1 ms delay, with a total irradiation time of 2 s, and approximate B1 field strength of 100 Hz. The selected frequency for the selective irradiation of protein was 0.5 ppm for on-resonance and 34 ppm for off-resonance. Correspondingly, free induction decay signals (FIDs) were subtracted automatically by phase cycling, providing the final 1D STD difference NMR spectra. The 90 readout pulse was followed by an optional T_2_ filter to reduce protein signals. The Watergate scheme from Varian was applied to selectively suppress the water signals. The total number of scans was 1024, and 16 ppm spectral widths were typically used for ^1^H STD spectra.

WaterLOGSY experiments were performed by using the same concentration as that in the STD NMR experiments. All spectra were acquired in 256 transients; 32,768 complex data points; 9842.52 Hz sweep width; 0.999947 s acquisition time; and 1.3 s mixing time. The spectra were collected on a Sun workstation using VnmrJ software (Varian package), and ACD/CNMR version 11.0 software (Advanced Chemistry Development Inc.) was used to analyze the spectrum data.

#### Molecular docking

The X-ray crystal structure of HSA was obtained from the Research Collaboratory for Structural Bioinformatics Protein Data Bank (PDB ID: 1H9Z; http://www.pdb.org). The 3D structure of MLN8237 was constructed from a 2D structure, and geometry was optimized using the “Discover Minimization” tool in Materials Studio 6.0 software. MGLTools 1.5.6 AutoDockTools with AutoGrid 4 and AutoDock 4 were used to set up and conduct docking calculations between MLN8237 and HSA. The PDB and PDBQT files of the receptor (HSA) and ligand (MLN8237) were prepared using MGLTools. The entire receptor was selected, all hydrogen atoms were added, and all water molecules and ions were removed. Docking was performed using the Lamarckian genetic algorithm implemented in AutoDock 4.2.5 to predict the binding site and type of interaction involved in the formation of MLN8237–HSA complex. The blind docking simulation strategy was adopted by setting the grid box size to 126 Å × 126 Å × 126 Å along the *x*-, *y-*, and *z-*axes, respectively, with 0.619 Å grid spacing, which covered the entire HSA molecule to allow the MLN8237 to explore all the possible binding sites of the protein. The center of the grid was set to the position of 23.758, 7.805, 16.725. After the grid map was generated, ligand flexible docking simulations (seven rotatable bonds) were performed with 100 runs; a moderate number of 250,000 energy evaluations; and a maximum of 27,000 generations. All other parameters were kept at their default values.

#### ITC titration

A MicroCal CN-ITC200 (MicroCal LLC, USA) titrator was used to obtain the thermodynamic information of binding between MLN8237 and HSA. The solutions were previously degassed using an ultrasonic device to prevent the formation of bubbles in the titrator cell. The MLN8237 solution (0.5 mM) in the syringe was titrated into the cell containing an HSA solution (0.023 mM) with successive injections. All titrations were performed at three different temperature levels (298, 304, and 310 K). After temperature equilibration, each successive injection was made into the reaction cell in 2 μL increments at 120 s intervals with stirring at 1000 rpm to ensure thorough mixing. The obtained data were analyzed using MicroCal-enabled Origin 7.0 software.

#### Fluorescence spectroscopic measurements

Fluorescence measurements were performed using a Cary Eclipse fluorescence spectrophotometer (Varian, USA) equipped with a 1.0 cm quartz cell. The spectra were recorded in a wavelength range of 290 to 450 nm with an excitation wavelength of 280 nm. The slit widths for excitation and emission were set to 5 and 10 nm, respectively. In the measurements, the concentrations of HSA were diluted to 2 μM, and MLN8237 concentrations were from 2 to 22 μM.

The synchronous fluorescence spectra of HSA with various MLN8237 concentrations were obtained at 298 K by considering the *D*-value between excitation and emission wavelengths (Δ*λ*) at 15 and 60 nm to determine the Tyr and Trp residues, respectively. For Δ*λ* = 15 nm, excitation wavelength was set from 260 to 310 nm. For Δ*λ* = 60 nm, excitation wavelength was set from 220 to 310 nm. The width of both excitation and emission slits were set at 5 nm and 10 nm. HSA concentration (2 μM) was fixed in the quartz cell, and different concentrations of MLN8237 were titrated into the solution. The 3D fluorescence spectra of HSA (2 μM) and HSA–MLN8237 solutions (1:2 and 1:4 ratios) were measured by using an excitation wavelength ranging from 200 to 400 nm with 5 nm increments and monitoring the emission spectra between 200 and 500 nm.

#### Circular dichroism (CD) spectra

Far UV-CD measurements (190–280 nm) were conducted on a Hitachi-F7000 fluorescence spectrometer equipped with a R3788 photomultiplier at 298 K that used a quartz cell of path length of 0.1 cm. HSA–MLN8237 ratios of 1:0, 1:1.3, and 1:2.6 M were obtained. Each CD spectrum was scanned at 60 nm min^−1^, and data points were recorded at 1 nm intervals for two-scan accumulations.

## Additional Information

**How to cite this article:** Yang, H. *et al*. Domain-specific interactions between MLN8237 and human serum albumin estimated by STD and WaterLOGSY NMR, ITC, spectroscopic, and docking techniques. *Sci. Rep.* **7**, 45514; doi: 10.1038/srep45514 (2017).

**Publisher's note:** Springer Nature remains neutral with regard to jurisdictional claims in published maps and institutional affiliations.

## Supplementary Material

Supplementary Information

## Figures and Tables

**Table 1:** Thermodynamic parameters for interaction of MLN8237 with HSA obtained from ITC at pH 7.4.

T (K)	*K* ( × 10^4^ M^−1^)	*N*	Δ*H* (kcal mol^−1^)	Δ*G* (kcal mol^−1^)	Δ*S* (cal mol^−1^ K^−1^)
298	12.3 ± 1.84	0.78 ± 0.05	−4.21 ± 0.38	−6.94	9.15
304	9.50 ± 2.28	0.84 ± 0.17	−3.58 ± 0.91	−6.92	10.98
310	7.79 ± 2.46	0.96 ± 0.11	−2.87 ± 0.43	−6.94	13.13

**Table 2:** 3D fluorescence spectral parameters of HSA alone and in the presence of MLN8237.

System	Peak No.	Peak Position [λ_ex_/λ_em_ (nm/nm)]	Stokes Shift ∆λ (nm)	Intensity
HSA	A	280/280 → 350/350	0	180.66 → 252.38
	1	280/335	55	546.96
	2	225/328	103	672.87
HSA-MLN8237 (1:2)	A	280/280 → 350/350	0	134.74 → 240.89
	1	280/336	56	390.35
	2	225/331	106	324.05
HSA-MLN8237 (1:4)	A	280/280 → 350/350	0	94.41 → 217.87
	1	280/336	56	295.05
	2	225/332	107	184.14
