# Supplementary material for: Domain-specific interactions between MLN8237 and human serum albumin estimated by STD and WaterLOGSY NMR, ITC, spectroscopic, and docking techniques
Source: Sci Rep. 2017 Mar 30;7:45514. doi: 10.1038/srep45514 (PMC5371984; doi:10.1038/srep45514)
Supplement: Supplementary Information [file srep45514-s1.pdf]

Supplementary Information

Domain-specific interactions between MLN8237 and human serum albumin estimated by STD and WaterLOGSY NMR, ITC, spectroscopic, and docking techniques

Hongqin Yang[1], Jiuyang Liu[2], Yanmei Huang[1], Rui Gao[2], Bin Tang[1], Shanshan Li[1], Jiawei He[1] & Hui Li[1*]

[1]College of Chemical Engineering, Sichuan University, Chengdu 610065, People's Republic of China.

[2]School of Life Sciences, University of Science and Technology of China, Hefei 230026, People's Republic of China.

*Corresponding Author: lihuilab@sina.com (Hui Li)

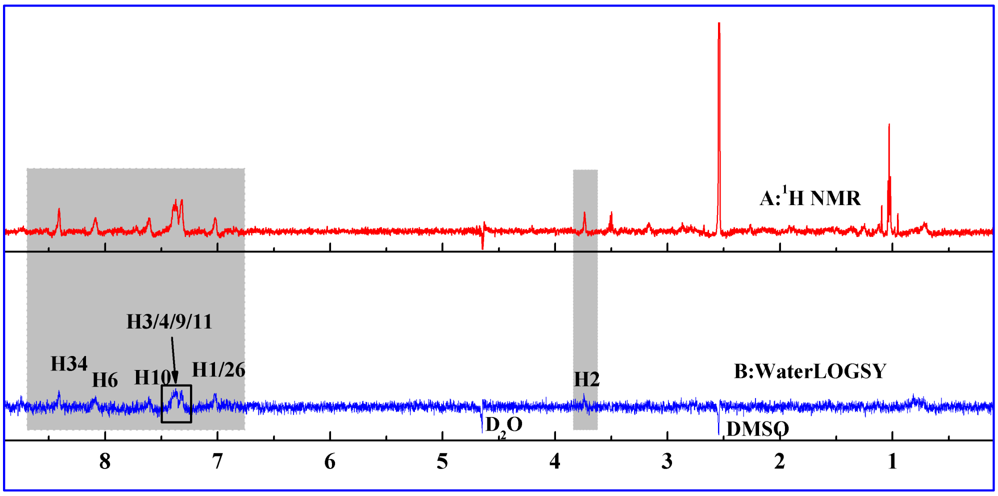

Supplementary Figure 1. ^{1}H NMR spectrum **(A)** and WaterLOGSY NMR spectrum **(B)** of 400 μM MLN8237 with 10 μM HSA (50% H_2O/50% D_2O PBS, pH 7.4 at 298 K). The D_2O and DMSO-d6 signals have been marked.

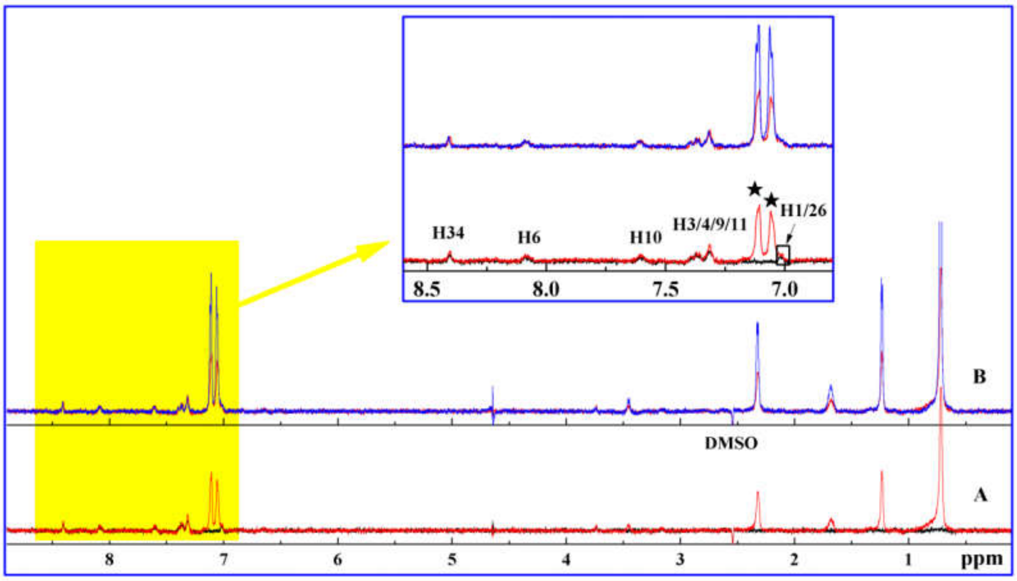

Supplementary Figure 2. STD competition binding experiments recorded at a 700 MHz spectrometer in 50% H_2O/50% D_2O PBS, pH 7.4 at 298 K. (A) Overlaid view of STD spectra of the system MLN8237 (400 μM)/HSA (10 μM) (black solid line) and MLN8237 (400 μM)/ibuprofen (480 μM)/HSA (10 μM) (red solid line); **(B)** Overlaid view of STD spectra of the system MLN8237 (400 μM)/ibuprofen (480 μM)/HSA (10 μM) (red solid line) and MLN8237 (400 μM)/ibuprofen (1200 μM)/HSA (10 μM) (blue solid line). The proton signals of ibuprofen are indicated by ★.

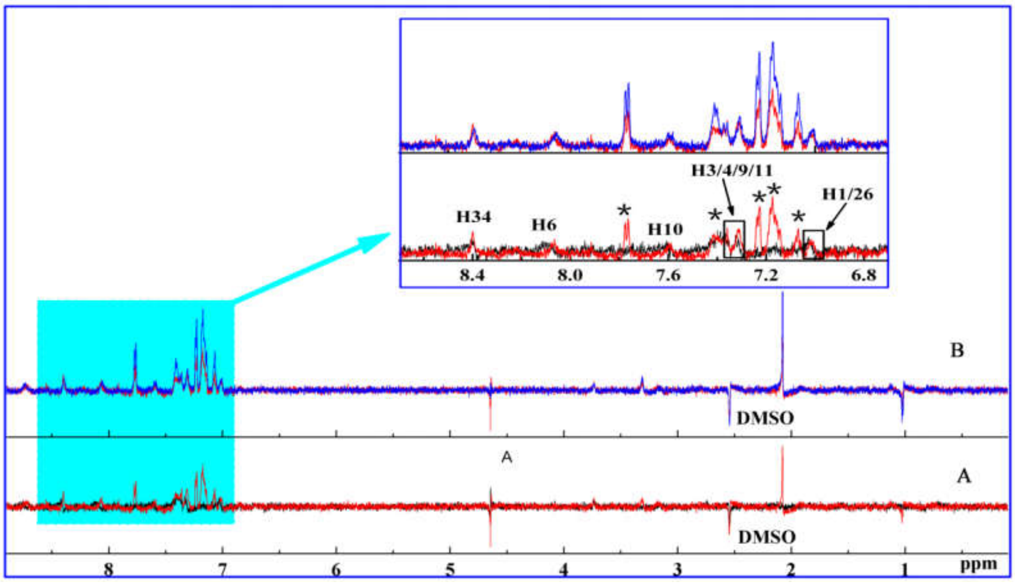

Supplementary Figure 3. WaterLOGSY competition binding experiments recorded at a 700 MHz spectrometer in 50% H_2O/50% D_2O PBS, pH 7.4 at 298 K. (A) Overlaid view of WaterLOGSY spectra of the system MLN8237 (400 μM)/HSA (10 μM) (black solid line) and MLN8237 (400 μM)/warfarin sodium (480 μM)/HSA (10 μM) (red solid line); **(B)** Overlaid view of WaterLOGSY spectra of the system MLN8237 (400 μM)/warfarin sodium (480 μM)/HSA (10 μM) (red solid line) and MLN8237 (400 μM)/warfarin sodium (1200 μM)/HSA (10 μM) (blue solid line). The proton signals of warfarin sodium are indicated by ＊.

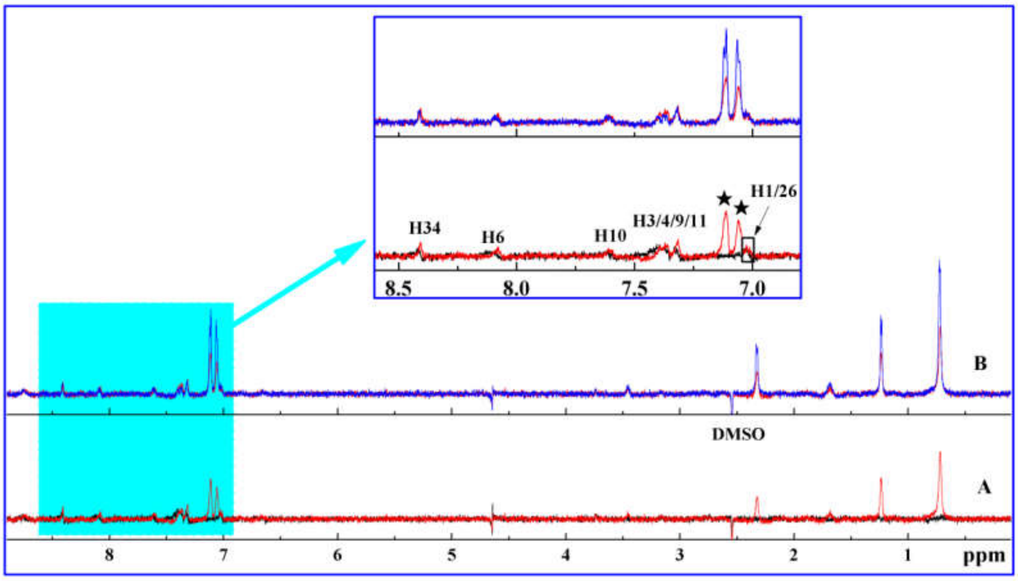

Supplementary Figure 4. WaterLOGSY competition binding experiments recorded at a 700 MHz spectrometer in 50% H_2O/50% D_2O PBS, pH 7.4 at 298 K. (A) Overlaid view of WaterLOGSY spectra of the system MLN8237 (400 μM)/HSA (10 μM) (black solid line) and MLN8237 (400 μM)/ibuprofen (480 μM)/HSA (10 μM) (red solid line); **(B)** Overlaid view of WaterLOGSY spectra of the system MLN8237 (400 μM)/ibuprofen (480 μM)/HSA (10 μM) (red solid line) and MLN8237 (400 μM)/ibuprofen (1200 μM)/HSA (10 μM) (blue solid line). The proton signals of ibuprofen are indicated by ★.

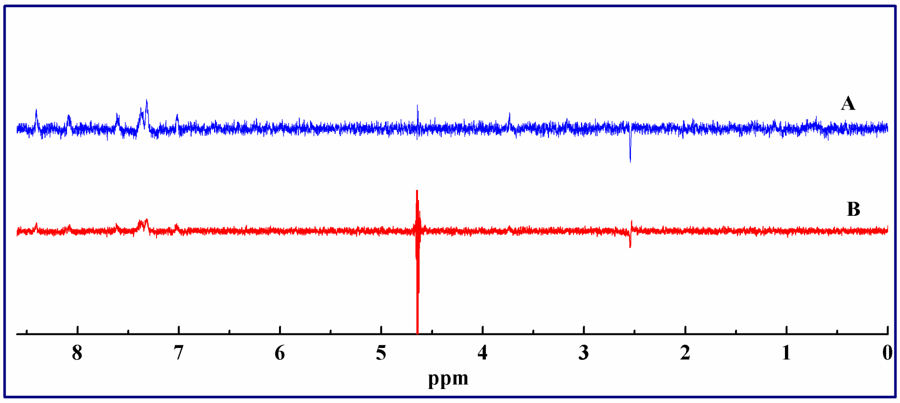

Supplementary Figure 5. (A) STD NMR spectrum of 50% H_2O/50% D_2O PBS, **(B)** STD NMR spectrum of 90% H_2O/10% D_2O PBS.

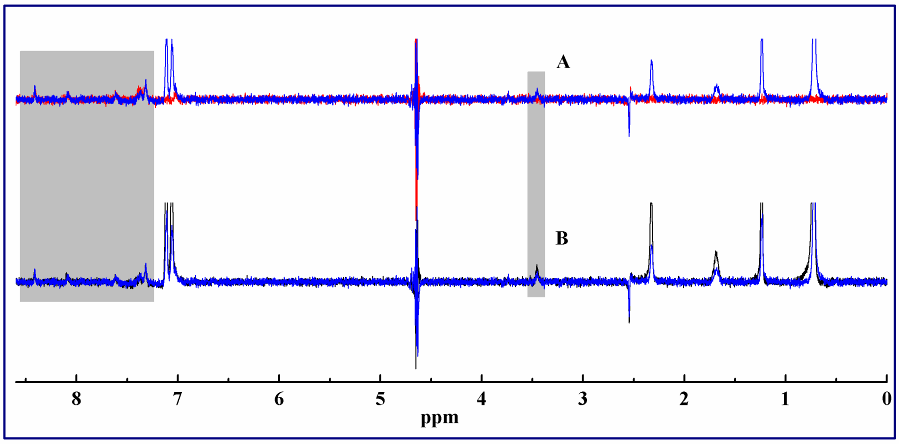

Supplementary Figure 6. STD competition binding experiments recorded at a 700 MHz spectrometer in 90% H_2O/10% D_2O PBS, pH 7.4 at 298 K. (A) Overlaid view of STD spectra of the system MLN8237 (400 μM)/HSA (10 μM) (red solid line) and MLN8237 (400 μM)/ibuprofen (480 μM)/HSA (10 μM) (blue solid line); **(B)** Overlaid view of STD spectra of the system MLN8237 (400 μM)/ibuprofen (480 μM)/HSA (10 μM) (blue solid line) and MLN8237 (400 μM)/ibuprofen (1200 μM)/HSA (10 μM) (black solid line).

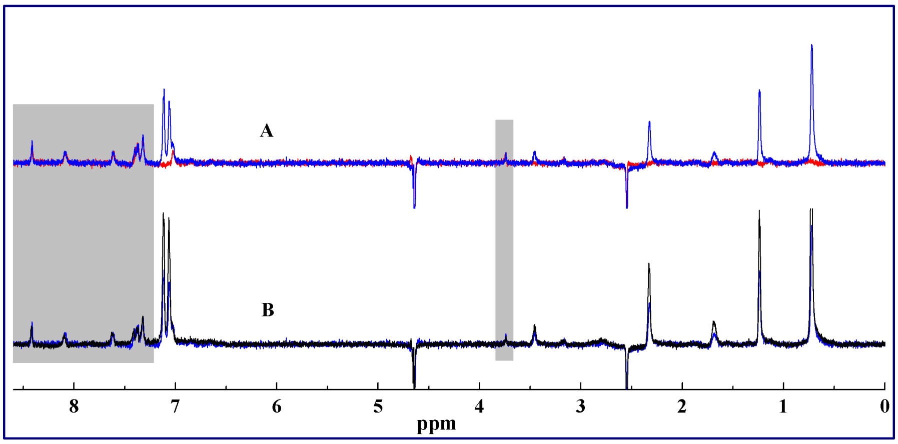

Supplementary Figure 7. WaterLOGSY competition binding experiments recorded at a 700 MHz spectrometer in 90% H_2O/10% D_2O PBS, pH 7.4 at 298 K. (A) Overlaid view of WaterLOGSY spectra of the system MLN8237 (400 μM)/HSA (10 μM) (red solid line) and MLN8237 (400 μM)/ibuprofen (480 μM)/HSA (10 μM) (blue solid line); **(B)** Overlaid view of WaterLOGSY spectra of the system MLN8237 (400 μM)/ibuprofen (480 μM)/HSA (10 μM) (blue solid line) and MLN8237 (400 μM)/ibuprofen (1200 μM)/HSA (10 μM) (black solid line).

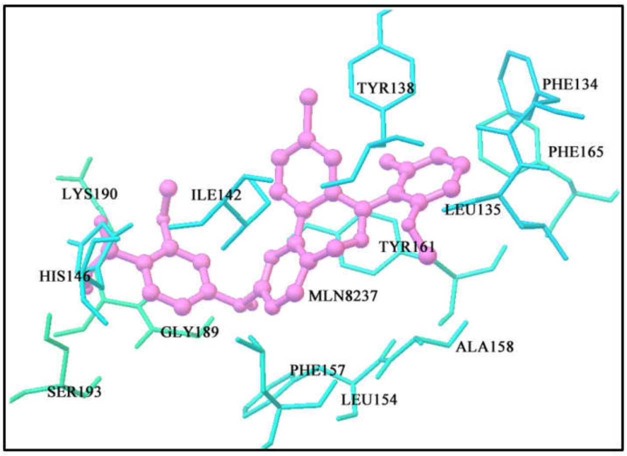

Supplementary Figure 8. The best docked result of the HSA-MLN8237 system. The MLN8237 structure is represented using a pink stick-and-ball model.

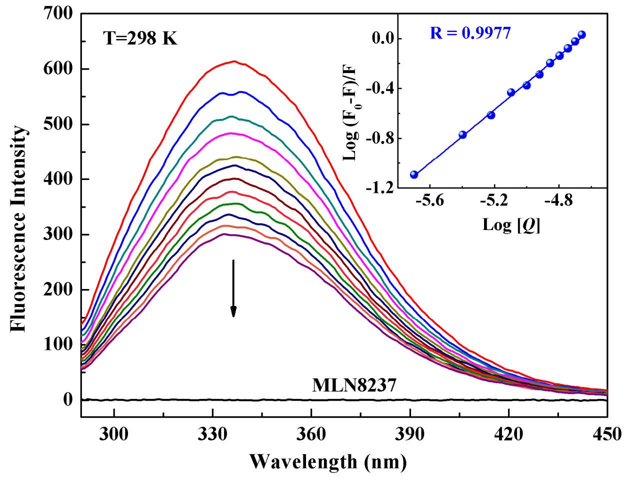

Supplementary Figure 9. Fluorescence spectra of HSA in the presence of MLN8237: [HSA] = 2 μM, [MLN8237] = 2, 4, 6, 8, 10, 12, 14, 16, 18, 20, 22 μM. Curve below shows the emission spectrum of 22 μM MLN8237 alone. The inset shows "modified" Stern-Volmer plot of the HSA-MLN8237 system at 298 K.
